# Delaying ripening using 1-MCP reveals chilling injury symptom development at the putative chilling threshold temperature for mature green banana

**DOI:** 10.3389/fpls.2022.966789

**Published:** 2022-09-15

**Authors:** Lan-Yen Chang, Steven A. Sargent, Jeongim Kim, Jeffrey K. Brecht

**Affiliations:** ^1^Division of Crop Improvement, Tainan District Agricultural Research and Extension Station, Tainan, Taiwan; ^2^Horticultural Sciences Department, University of Florida, Gainesville, FL, United States

**Keywords:** texture analysis, vascular browning, chlorophyll fluorescence, electrolyte efflux, shelf-life limiting factor

## Abstract

Storage at the putative chilling threshold temperature (CTT) to avoid chilling injury still limits postharvest handling of tropical fruit like banana in that ripening may occur at the CTT. To determine whether chilling injury (CI) symptoms would develop in mature green (MG) banana fruit if the CTT exposure was extended by inhibiting ethylene action and thus ripening, 1-methylcyclopropene (1-MCP) was applied. Individual ‘fingers’ from multiple ‘clusters’ of MG bananas were either immersed in water or 50 μg L^−1^ 1-MCP (a.i.) solution and each treatment was divided into three subgroups for storage at 5.0°C (severe CI), 13.0°C (mild CI), or 14.0°C (CTT) ± 0.1°C. 1-MCP delayed ripening in terms of color change for 10 days for fruit stored at the CTT. Ethylene production by fruit at 5.0°C remained around 0.04 ng kg^−1^ s^−1^ with no obvious increase during 31-day storage. Ethylene production at 14.0°C (−1-MCP/+1-MCP) increased on Day 33 while increasing on Day 38 for 13.0°C fruit without 1-MCP and on Day 39 for fruit with 1-MCP. Peak climacteric ethylene occurred on Days 44 and 39 for 13.0 and 14.0°C fruit without 1-MCP, respectively, and on Days 59 and 51 for 13.0°C and 14.0°C 1-MCP-treated fruit, respectively. As hypothesized, longer exposure of MG banana fruit to the CTT of 14.0°C without onset of ripening as was allowed by prior 1-MCP treatment allowed CI to develop at that normally non-chilling temperature. Vascular browning was the first visual and most sensitive CI symptom in the experiment and was observed on Day 4 at 5.0°C, Day 10 at 13.0°C, Day 19 at 14.0°C without 1-MCP, and on Day 28 at 14.0°C with 1-MCP. Using a 1-MCP pre-treatment to remove the influence of ethylene from bananas stored at 13°C or 14°C also resulted in slight reduction in vascular browning severity. In conclusion, a putative safe temperature may become a CI temperature if the shelf-life-limiting factor is removed, allowing longer exposure. Chilling at the CTT caused relatively mild injury on fruit, and vascular browning is a sensitive indicator of CI status, while the light-adapted quantum yield of photosystem II [Y(II)] could be a non-destructive indicator of early CI stress in MG banana. Fruit at 13.0/14.0°C developed CI symptoms slightly later with 1-MCP than without 1-MCP. This suggests that ethylene might be involved in early CI symptom development.

## Introduction

Temperature management is essential to control crop metabolic activity and therefore maintain the quality of a commodity for the longest shelf-life before deterioration from senescence or decay occurs. However, most tropical crops, which are chilling sensitive, can be dramatically and negatively affected by low-temperature storage. Below some temperature limit, physiological damage, known as chilling injury (CI), occurs in plant tissues (Lyons, 1973). The temperature limit, or chilling threshold temperature (CTT) is a practical term within the produce industry for the lowest safe storage or transport temperature at which CI is never encountered under usual postharvest handling conditions. Therefore, for climacteric tropical fruit species, the major shelf-life limitation is CI at chilling temperatures and ripening at non-chilling temperatures. Banana (*Musa* spp., AAA group, Cavendish type), one of the most economically important horticultural crops worldwide, is a chilling-sensitive, climacteric tropical fruit requiring strict temperature control [recommended storage temperature range from 13.3 to 14.4°C (Thompson, 2011)] to maintain its commercial value. Classical CI symptoms of banana fruit include peel surface discoloration, subepidermal vascular browning, delayed or abnormal ripening, and sometimes failure to ripen, which are related to both temperature and exposure time. Underpeel discoloration, the industry term for vascular browning, is the earliest visible symptom of banana CI (Harvey, 2005), and reputedly can result from exposing mature-green (MG) banana to 1 h at 10°C, 5 h at 11.7°C, 24 h at 12.2°C, or 72 h at 12.8°C (P.E. Brecht, PEB Commodities, formerly Corp. Dir. Qual. Control, United Brands/Chiquita, personal communication). However, the alteration of membrane lipids is generally regarded as the first step of CI response in plant cells and precedes the appearance of visible CI symptoms (Marangoni et al., 1996).

Low temperature is perceived by plants as a kind of stress. When plant tissues are exposed to an injurious low temperature, the membrane conformation and structure are firstly affected, and parameters related to membrane permeability, such as electrolyte efflux (EE) and lipid oxidation, composition adjustment, or phase transition of membrane lipids, proceed as the severity of chilling increases (Lyons, 1973; Saltveit, 1991). However, the longer the duration of chilling exposure or the lower the temperature during exposure, the more severe the CI symptoms that were observed in lemon (*Citrus limon* L. cv. Eureka; Eaks, 1980), avocado (*Persea americana* cv. ‘Hass’; Eaks, 1983), and banana (Trejo-Márquez and Vendrell, 2010). As CI and severe CI symptoms are irreversible, cells probably die in the extreme condition.

Ripening-related ethylene production by climacteric fruits like banana is triggered by maturation stage, controlled by specific silent genes (Yang and Hoffman, 1984; Manning et al., 2006; Lü et al., 2018). Ethylene production is also often regarded as being involved in the response of plants to various stresses, including chilling, freezing, high temperature, and mechanical injury. The ethylene produced by fruit tissues in response to chilling stress could play a role in the development of CI or CI symptoms. Some fruit such as zucchini (*Cucurbita pepo* L.) have been reported to produce increased amounts of ethylene as storage duration below the CTT is extended (Megías et al., 2016); some fruit produce a great amount of ethylene upon rewarming from the chilling temperature (e.g., cucumber, *Cucumis sativus* L.; Eaks and Morris, 1956; Andersen and Kent, 1982). For banana, Li et al. (2015) applied ethylene to mature-green (MG) banana fruit before exposure to chilling conditions and suggested that the ethylene alleviated CI symptom development. However, the ethylene application also accelerated the ripening process, which would have likely reduced CI sensitivity as shown for tomatoes (Chomchalow et al., 2002; Biswas et al., 2014), since banana fruit are extremely ethylene-sensitive. It has been reported that the ripening process in physiologically MG banana fruit can be triggered by 0.015 to 0.5 μL L^−1^ ethylene (Marriott and Palmer, 1980; Chang and Hwang, 1990).

1-Methylcyclopropene (1-MCP) is an ethylene action inhibitor that effectively binds irreversibly to ethylene receptors, preventing the signal transduction regulating ethylene action (Sisler et al., 1996a). It has been found that 1-MCP treatment inhibited the development of CI in avocado (Pesis et al., 2002), pineapple (*Anana comosu*s L. Merr; Selvarajah et al., 2001), and plum (*Prunus salicina* L.; Candan et al., 2006); however, 1-MCP was reported to exacerbate CI symptoms in “Empire” apple (*Malus domestica* Borkh; Watkins, 2008). These results suggest that the response of different species of fruit to ethylene under chilling stress varies and that the mechanisms of ethylene action on CI and its development in various harvested fruit are relatively complex.

Since banana shelf life is limited by ripening at the CTT, application of ethylene antagonists such as 1-MCP could be a promising practice to delay the onset of the ripening process and thus extend the shelf life. However, the resulting extension of banana shelf-life at the CTT by 1-MCP application raises the possibility that CI could replace ripening as the shelf-life limiting factor. That is because the longer-than-normal exposure to the slightly higher temperature that would be made possible by 1-MCP treatment might allow CI to occur that is usually obviated by ripening.

The basis for conducting the research being reported here was related to the following two suppositions: first, that tropical climacteric fruit shelf life is limited by CI at chilling temperatures and by ripening at non-chilling temperatures; second, that the ethylene produced by fruit tissues in response to chilling stress could play a role in the development of CI. We used 1-MCP treatment prior to storage of MG bananas at their CTT (i.e., their lowest non-chilling temperature of 14.0°C) or at a chilling temperature just below the CTT (i.e., 13.0°C) to explore those two suppositions. For comparison, we also stored bananas at the extreme chilling temperature of 5.0°C.

The objectives of this study were to determine the possible role of ethylene in the development of banana CI and/or CI symptom development by using 1-MCP to remove ethylene involvement, and to examine the potential chilling stress parameters that might be affected by ethylene.

## Materials and methods

### Sample preparation and treatments

MG banana fruit (*Musa* spp., AAA group Cavendish subgroup) without exogenous exposure to ethylene were obtained through a local retailer in Florida directly after receipt at the retailer’s local distribution center, and about 4–5 days after harvest in Guatemala, with the bananas having been maintained at 13.5–14.0°C, depending on the season. Once received at a local store in Gainesville, the fruit were transferred to the Postharvest Laboratory, University of Florida, FL, United States. To reduce variation in maturity, clusters with a* < −14 and hue angle (h^*^) < 105 ° were selected from a group representing twice the number required for each experiment. Selected clusters (i.e., “hands”) were divided into individual fruit (i.e., “fingers”), selecting only those at stage 2 (1–7 scale; von Loesecke, 1950; Supplementary Figure 1) and discarding fruit with nonuniform size and color, or with defects. The selected fingers were randomized and then washed and sanitized using 100 μL L^−1^ peroxyacetic acid for 3 min and allowed to air dry before further treatment. The experiment described here was conducted twice using fruit obtained and handled in this way with similar results and representative results are presented.

Preparation of aqueous 1-MCP solution was based on Choi and Huber (2008) and Hagan et al. (2017). Solutions were prepared with 1-MCP powder (AF × RD-380, AgroFresh, Inc., Rohm and Haas, Philadelphia, PA, United States) at 0 or 50 μg L^−1^ (active ingredient; a.i.), with the powder suspended in 10 L of distilled water in a 20 L plastic bucket by stirring for 1 min. Based on our preliminary measurements of 1-MCP release kinetics, we determined that loss of 1-MCP due to volatilization was minimal if the 1-MCP solution was used less than 45 min after preparation. Therefore, within 10 to 45 min following preparation, banana fingers for the two treatments were immersed in the solutions for 60 s at 23.0°C followed by air drying. The immersion and drying of the bananas for the two treatments were done separately to avoid 1-MCP cross-contamination. After drying, the control and 1-MCP bananas were stored overnight in separate rooms at 14.0°C and 95% relative humidity (RH).

On the following day, bananas treated with 0 or 50 μg L^−1^ aqueous 1-MCP (a.i.) solutions were then divided into three subgroups (51 fingers per subgroup plus an additional approximately 10% fruit for observations) and transferred to 5°C (severe CI), 13°C (mild CI), or 14°C (CTT; ± 0.1°C) and 95% RH (± 1%) in the darkness. The customized Conviron controlled temperature rooms (Controlled Environments Ltd., Winnipeg, Manitoba, Canada) that were used for these experiments maintained the setpoint temperature ± 0.1°C using a hot gas bypass system and continuous compressor and fan operation; the RH was maintained at ±1% by a dry fog system (ES100, Smart Fog Inc., Reno, NV, United States).

### Ripeness stage

Three randomly selected banana fingers within the same treatment were selected and their ripeness stage, from 2 (light green) to 7 (yellow with brown spots), was judged visually by the external skin color using a standard banana color chart (von Loesecke, 1950; Supplementary Figure 1). The ripeness stages of the same three banana fingers per treatment were reevaluated at each subsequent sampling time.

### Respiration rate and ethylene production measurement

Respiration and ethylene production were measured every 4 h during the first 3 d, then every day until Day 9, then every 2 d during Days 11–37, and back to daily until the last measurement (up to 61 days depending on the treatment). Three replicates of three banana fingers from each treatment were maintained in 1.75 L glass containers equipped with lids with rubber septa and were sealed for 1 h at 13°C or 14°C and 1.5 h for 5°C. A 3.0-mL headspace sample was withdrawn with a syringe for CO_2_ and ethylene measurements using a Varian CP-3800 gas chromatograph (Varian Inc., Walnut Creek, CA, United States) equipped with a thermal conductivity detector (TCD) and a pulse discharge helium ionization detector (PDHID) as described in detail by Chang and Brecht (2020b). The carrier gas (helium) flow rate was 0.33 mL s^−1^. The injector and columns were operated at 220°C and 50°C, respectively. The PDHID was operated at 120°C and the TCD was operated at 130°C.

### Texture analysis

The texture of the peel and pulp of three banana fingers per treatment was determined by using a texture analyzer (TA.XT plus, Texture Technologies Corp. and Stable Micro Systems, Ltd., Hamilton, MA, United States) every 3 d beginning on Day 1, except for the last three evaluations, which were conducted on Days 43, 51, and 61. Samples were prepared after equilibrating to room temperature (23°C). For peel texture analysis, banana peel from near the equatorial region was cut into two, 2.5 × 2.5 cm squares per finger. Banana peel shear resistance was evaluated by putting the epidermal side of the peel tissue downward and using a 50-kg load cell fitted with a 3-mm thick stainless steel flat-edge blade positioned parallel to the fiber direction. The peel was then sheared to a depth of 5 mm at a rate of 10 mm min^−1^ and the maximum load force was recorded and expressed in Newton (N). Pulp texture analysis was performed on two 5-cm-thick cross sections of pulp tissue sliced from whole fruit near the equatorial region. The measurement was performed using a 50-kg load cell fitted with an 8-mm stainless steel Magness–Taylor probe (convex tip), which was positioned at zero-force contact on the equatorial region of the sample, positioned horizontally on its side on a solid platform. Pulp slices were compressed to a depth of 10 mm at a rate of 10 mm min^−1^. The maximum load force over the distance of the probe travel was recorded and expressed in Newton (N).

### Evaluation of peel graying, vascular browning, and chlorophyll fluorescence

Chilling injury severity in banana fruit was scored visually on three banana fingers per treatment every 3 day beginning on Day 1, except for the last three evaluations, which were conducted on Days 43, 51, and 61. Peel graying was determined by estimating the percentage area of banana peel turning gray or grayish-brown. Vascular browning in the peel aerenchyma tissue was expressed as percentage area of browning after removing the peel epidermal layer from the middle portion (*ca.* 3 × 5 cm^2^) of a finger using a potato peeler (Supplementary Figure 2).

Chlorophyll fluorescence was measured every half-day during the first 3 d, then every day until Day 9, then every 2 d until the last day at each treatment temperature (5 ± 0.1, 13 ± 0.1, or 14 ± 0.1°C). Photosystem II (PSII) function of chloroplasts was determined through the modulated chlorophyll fluorometer OS5p + (Opti-Science Ltd., Hudson, United States). For the dark-adapted test of maximum quantum yield (photochemical efficiency of PSII), the fruit were placed in continuous dark for at least 30 min and the equatorial region of samples was evaluated in the dark without light disruption while collecting data of Fv/Fm. For the light-adapted test on the quantum yield of PSII, fruit samples were exposed to illumination from a full spectrum LED light fixture (40 W, 5000 K, 4100 lumens, Sunco Lighting, United States) for at least 30 min and the equatorial region of the sample exposed to light evaluated using Y(II) mode.

### Peel electrolyte efflux and malondialdehyde content

Fruit samples were evaluated every 6 days, except for the last three intervals at Days 43, 51, and 61. Electrolyte efflux (EE) of banana peel was evaluated using peel tissue disks (*n* = 3) excised from the equatorial region using a 9-mm diameter (No. 5) cork borer. Disks of each fruit peel sample were rinsed with distilled water followed by drying with paper towels and immersion in 10 mL of 0.7 M mannitol solution at 23.0°C for 4 h (Villalta and Sargent, 2004) with gentle shaking. The conductivity of the mannitol bathing solution after 4 h incubation was measured using a YSI-31A conductivity meter (Model 3,403, Yellow Springs, OH, USA). Total electrolyte content was determined after freezing the tissue samples at −20°C for at least 24 h. Frozen samples were thawed and boiled for 30 min and the conductivity of the bathing solution was re-measured. Electrolyte efflux was expressed as a percentage of total tissue electrolyte content.

Malondialdehyde (MDA) measurement was done as described by Huang et al. (2013). Frozen peel tissue (2.0 g) was homogenized in 10 mL of 100 g L^−1^ trichloroacetic acid and centrifuged at 10,000 × *g_n_* for 20 min. A 1-mL aliquot of the resulting supernatant was collected and mixed with 3 mL of 5 g L^−1^ thiobarbituric acid. The mixture was boiled for 15 min, cooled in an ice bath, and centrifuged at 12,000 × *g_n_* for 15 min. The clear supernatant was collected and used to measure absorbance at 450, 532, and 600 nm. The MDA concentration was calculated according to the formula: 6.453 × (A532 − A600) − 0.563 × A450. The concentration of MDA on a fresh weight basis was calculated as mmol kg^−1^.

### Statistical analysis

The experimental design was a two-factor repeated measurement completely randomized design. Data were subjected to repeated measures analysis of variance (RM-ANOVA) using JMP statistical software (Version 8, SAS Institute, Cary, NC, USA). Fisher’s least significant differences (LSD, *p* ≤ 0.05) were determined to compare differences between treatment means following identification of a significant ANOVA effect. The experiment reported was conducted two times and representative results presented.

## Results

### Ripening

The repeated experiments gave statistically similar results; therefore, only the data of the second experiment are presented here. Fruit stored at 5.0°C did not ripen during the 31-day storage period and the peel turned to dark brown after 16 days whether treated with 50 μg L^−1^ 1-MCP or not (data not shown). The fruit in the remaining treatments were maintained at stage 2–2.5 until at least Day 34 and only the greenness of the peel faded slowly and the h* decreased slightly from 115° to 110° during the first 30 days for the fruit stored at 13.0/14.0°C (color data not shown). Fruit without 1-MCP treatment (−1-MCP) stored at 13.0 or 14.0°C “turned” (appearance of yellow color; initiation of ripening) after 45 or 36 days, respectively (Table 1). The drastic decline of the h^*^ corresponded to the ripeness stage and occurred first in the −1-MCP fruit at 14.0°C and then in the −1-MCP fruit at 13.0°C. Fruit treated with 1-MCP (+1-MCP) and stored at either 13.0 or 14.0°C started to ripen after 57.7 and 53 days, showing little difference in appearance. On the last day of the experiment (Day 61), fruit from the −1-MCP treatments stored at 13.0/14.0°C had reached color stage 6 or 7 while fruit from the +1-MCP treatments were still at color stage 3.5 or 4. Thus, the 1-MCP treatment delayed the ripening process significantly.

**Table 1:** Changes in ripeness stage of banana fruit that were initially treated with 0 (−1-MCP) or 50 μL L^−1^ (+ 1-MCP) aqueous 1-MCP for 60 s at 23.0°C and then transferred to 5.0°C, 13.0°C or 14.0°C storage with 95% RH (*n* = 3).

Storage temperature (°C)	Treatment	Initial ripeness stage	Average days to initiate the ripening process (obvious color changes)	Average color stage at the end of the experiment (Day 61)^*^
5. 0	− 1-MCP	2.17a^**^	–^*^	–
	+ 1-MCP	2.17a	–^*^	–
13.0	− 1-MCP	2.23a	45c	5.6b (Green tip to fully yellow)
	+1-MCP	2.23a	57.7a	4c (Yellow more than green)
14.0	− 1-MCP	2.17a	36d	7a (Fully yellow with brown spots)
	+ 1-MCP	2.17a	53b	3.5c (Green more than yellow to yellow more than green)

* Banana fruit stored at 5.0°C turned brown and were discarded after 31 days. Before then, there were no ripening characteristics observed.

** Different letters in the same column indicate significant differences by treatments.

### Respiration and ethylene production

Respiration (CO_2_ production) and ethylene production exhibited similar patterns during storage. The initial respiration rate of the fruit at 14.0°C was 1.4 μg kg^−1^ s^−1^. After transfer to the severe chilling treatment (5.0°C), respiration rate declined to 0.8–0.9 μg kg^−1^ s^−1^ and remained constant (Table 2). Fruit stored at 13.0 or 14.0°C maintained a similar respiration rate about 1.28 or 1.35–1.41 μg kg^−1^ s^−1^ for first 29 days. The first 3 days were intensively examined in order to detect irreversible CI development, but no significant stress-related respiratory burst was observed. The critical climacteric respiration characters are shown in Table 2. Fruit in the 14.0°C/−1-MCP treatment began firstly to increase respiration rate after 29 days, followed by fruit in the 13.0°C/−1-MCP treatment after 35 days. The increase in respiration rate for 1-MCP-treated fruit was delayed. Fruit at 14.0°C/+1-MCP were observed to commence the respiratory climacteric after 48 days while at 13.0°C/+1-MCP the respiration rate began to rise on Day 54. The peak climacteric respiration rate was also affected, reaching 8.85 μg kg^−1^ s^−1^ on Day 43 for the 14.0°C/−1-MCP fruit, 7.0 μg kg^−1^ s^−1^ around Day 51 for the 13.0°C/−1-MCP fruit, 6.29 μg kg^−1^ s^−1^ after 58 days for the 14.0°C/+1-MCP fruit, and 6.0 μg kg^−1^ s^−1^ on Day 56 for the 13.0°C/+1-MCP fruit.

**Table 2:** Changes in respiration rate (CO_2_ production) of banana fruit that were initially treated with 0 (−1-MCP) or 50 μL L^−1^ (+ 1-MCP) aqueous 1-MCP for 60 s at 23.0°C and then transferred to 5.0°C, 13.0°C or 14.0°C storage with 95% RH (*n* = 3).

Storage temperature (°C)	Treatment	Average respiration rate (μg kg^−1^ s^−1^) during the first 3 days	Days to the climacteric rise of respiration	Days to the climacteric peak of respiration	Average respiration rate (μg kg^−1^ s^−1^) at the climacteric peak	Average respiration rate (μg kg^−1^ s^−1^) at the end of the experiment (day 31 at 5°C; day 61 at 13/14°C)^*^
5. 0	− 1-MCP	0.85d^**^	–^*^	–^*^	–^*^	1.55
	+ 1-MCP	0.91d	–^*^	–^*^	–^*^	1.89
13.0	− 1-MCP	1.28c	35.7c	51.3c	7.01b	6.49a
	+ 1-MCP	1.28c	54a	60.8a	7.49ab	7.22a
14.0	− 1-MCP	1.35b	29.7d	43.7d	8.85a	6.99a
	+ 1-MCP	1.41a	48b	58.5b	6.29b	4.64b

* Banana fruit stored at 5.0°C turned brown and were discarded after 31 days. Before then, there were no ripening characteristics observed.

** Different letters in the same column indicate significant differences by treatments.

In terms of ethylene production, all fruit produced around 0.04 ng kg^−1^ s^−1^ before being initially transferred from 14.0°C to the different treatment temperatures (Table 3). The ethylene production rate of fruit stored at 13.0°C/14.0°C − 1-MCP began to increase after 37 days, following by 14.0°C/+1-MCP (42 days) and 13.0°C/+1-MCP (46 days). The climacteric peak of ethylene production was 0.16–0.23 ng kg^−1^ s^−1^ on around Day 53–55 for fruit in the 13.0°C, 14. 0°C/−1-MCP, and 14.0°C/+1-MCP treatments, and on Day 58 for the 13.0°C/+1-MCP treatment.

**Table 3:** Changes in ethylene production rate of banana fruit that were initially treated with 0 (−1-MCP) or 50 μL L^−1^ (+1-MCP) aqueous 1-MCP for 60 s at 23.0°C and then transferred to 5.0°C, 13.0°C or 14.0°C storage with 95% RH (*n* = 3).

Storage temperature (°C)	Treatment	Average ethylene production (ng kg^−1^ s^−1^) during the first 3 days	Days to the climacteric rise of ethylene production	Days to the climacteric peak of ethylene production	Average ethylene production (ng kg^−1^ s^−1^) at the climacteric peak	Average ethylene production (ng kg^−1^ s^−1^) at the end of the experiment (day 31 at 5°C; day 61 at 13/14°C)^*^
5. 0	−1-MCP	0.036b^**^	–			0.032
	+1-MCP	0.040a	–			0.037
13.0	−1-MCP	0.035b	38.7b	54b	0.166c	0.136c
	+1-MCP	0.034b	46a	58a	0.338a	0.301a
14.0	−1-MCP	0.032c	37b	53.7b	0.214bc	0.145bc
	+1-MCP	0.036b	42.2ab	55.3ab	0.232b	0.188b

* Banana fruit stored at 5.0°C turned brown and were discarded after 31 days. Before then, there were no ripening characteristics observed.

** Different letters in the same column indicate significant differences by treatments.

### Textural analysis of banana peel and pulp

The textural changes in the banana peel and pulp were asynchronous. Resistance of banana peel to shear force for the fruit stored at 5.0°C remained within the range of 100 to 120 N during the entire 31-day storage period while the peel shear resistance of the fruit at 13.0/14.0°C increased from 100 to 140 N over the same period (Table 4). After 34 days, the peel shear resistance for the −1-MCP treatments stored at 13.0 or 14.0°C began declining, falling below 100 N by Day 51 and Day 37, respectively, while the 13.0 or 14.0°C/+1-MCP fruit retained a peel shear resistance of approximately 100 N throughout storage. Peel shear resistance was affected mostly by temperature before 31 days, but 1-MCP was the major factor determining peel shear resistance after 34 days.

**Table 4:** Changes in peel resistance to shear force (*N*) and pulp firmness (*N*) of banana fruit that were initially treated with 0 (−1-MCP) or 50 μL L^−1^ (+1-MCP) aqueous 1-MCP for 60 s at 23.0°C and then transferred to 5.0°C, 13.0°C or 14.0°C storage with 95% RH (*n* = 3).

Storage temperature (°C)	Treatment	Initial banana peel resistance to shear force (N) on the first day	Days to peel resistance to shear force below 100 *N*	Banana peel resistance to shear force (*N*) at the end of the experiment	Initial banana pulp firmness (*N*) on the first day	Days to the pulp firmness below 20 *N*	Banana pulp firmness (*N*) at the end of the experiment (day 31 at 5°C; day 61 at 13/14°C)^*^
5.0	−1-MCP	100.68d^**^	–	107.21^*^	34.11a	–	34.11
	+1-MCP	100.74d	–	119.13^*^	31.73a	–	31.73
13.0	−1-MCP	107.34bc	51	43.17b	34.11a	42ab	1.66a
	+1-MCP	119.34a	-	119.79a	31.73a	51a	1.92a
14.0	−1-MCP	111.78b	37	47.29b	34.11a	37b	1.82a
	+1-MCP	111.69b	-	106.72a	31.73a	42ab	1.65a

Banana peel resistance to sheer force (*N*) with the epidermis oriented downward and the fiber direction oriented parallel to a 3-mm diameter flat blade and banana pulp firmness (*N*) measured using a 8-mm diameter Magness-Taylor probe.

* Banana fruit stored at 5.0°C turned brown and were discarded after 31 days. Before then, there were no ripening characteristics observed.

** Different letters in the same column indicate significant differences by treatments.

Firmness of banana pulp was initially 31–34N (Table 4). Pulp firmness of fruit stored at 5.0°C (+1-MCP/−1-MCP) remained constant throughout storage while the pulp firmness in the other treatments declined beginning approximately on Day 34 (data not shown). Pulp firmness in the −1-MCP treatments at 13.0/14.0°C and + 1-MCP treatment at 14.0°C declined drastically to below 20N after 37–42 days while the +1-MCP/13.0°C fruit softened more gradually to the same firmness over 51 days.

### Gray peel and vascular browning

Results for visual CI symptoms are presented in Table 5. Only fruit stored at 5.0°C (+1-MCP/−1-MCP) exhibited gray skin development, a symptom of CI, which turned to dark brown as storage proceeded. The other treatments did not show significant external discoloration. Storage temperature was the major factor controlling gray peel discoloration, and only during the 31 days of storage duration for the 5.0°C treatments.

**Table 5:** Chilling injury symptom development on banana fruit that were initially treated with 0 (−1-MCP) or 50 μL L^−1^ (+1-MCP) aqueous 1-MCP for 60 s at 23.0°C and then transferred to 5.0°C, 13.0°C or 14.0°C storage with 95% RH (*n* = 3).

Storage temperature (°C)	Treatment	Average days to show the first sign of gray peel (percentage area observed)	Average days to show the first sign of vascular browning (percentage area observed)	Average percentage vascular browning area observed at the end of the experiment (day 31 at 5°C; day 61 at 13/14°C)^*^
5.0	−1-MCP	4a^**^^,^^***^ (40%)	4d (16.7%A)	95
	+1-MCP	4a (40%)	4d (16.7%A)	95
13.0	−1-MCP	–^***^	10c (8.3%B)	15
	+1-MCP	–^***^	10c (2.3%C)	10
14.0	−1-MCP	–^***^	19b (1%C)	8.3
	+1-MCP	–^***^	28a (8.7%B)	10

* Banana fruit stored at 5.0°C suffered severe chilling injury turning brown and were discarded after 31 days. Before then, there were no ripening characteristics observed.

** Different letters in the same column indicate significant differences by treatments.

*** There was no gray peel observed on banana fruit stored at 13 or 14°C.

Fruit stored at 5.0°C (+1-MCP/−1-MCP) developed brown streaks from discoloration of vascular strands in the aerenchyma tissue of the peel after 4 days and this browning area in vascular tissues kept increasing to 100% on Day 20 (data not shown). Treatments at 13.0°C (+1-MCP/−1-MCP) showed some brown spots and streaks after 10 days, but the severity was reduced by 1-MCP treatment so that around 3% area was affected for the +1-MCP fruit compared with around 8% for the −1-MCP fruit. The −1-MCP fruit at 14.0°C developed vascular browning after 19 days while the +1-MCP fruit required 28 days to develop vascular browning. Vascular browning area did not exceed 10% throughout the experiment for fruit stored at 13.0/14.0°C.

### Chlorophyll fluorescence

**Figure 1:**
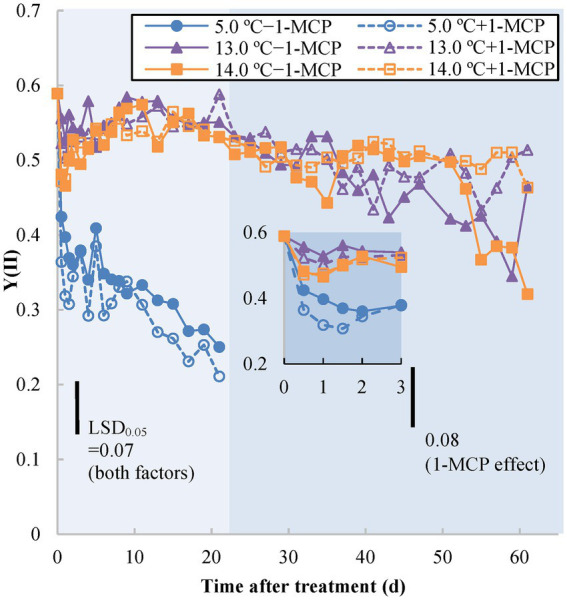
Photochemical efficiency as measured by light-adapted status photosystem II chlorophyll fluorescence quantum yield [Yield (II)] of banana peel initially treated with 0 (−1-MCP) or 50 μL L^−1^ (+ 1-MCP) aqueous 1-MCP for 60 s at 23.0°C and transferred to 5.0, 13.0 or 14.0°C storage with 95% RH (*n* = 3). LSD_0.05_ values varied within different time frames shown by different background shading (days 0–21; days 23–61) due to the various shelf-life durations of treatments.

Yield (II) [Y(II)], known as the quantum yield of photosystem II (PSII) at light-adapted status, indicates operating efficiency of PSII under real-time environmental conditions. The initial Y(II) value on Day 0 was 0.6 (Figure 1). The Y(II) value of fruit stored at 5.0°C (+1-MCP/−1-MCP) rapidly decreased within the first 0.5 d and thereafter continued declining more slowly, reaching 0.2 to 0.3 at the end of observation (Day 21; see insert in Figure 1). The Y(II) values of fruit at 13.0/14.0°C and + 1-MCP/−1-MCP changed little during the first 5 weeks of storage. Beginning around Day 37, the Y(II) of the 13.0°C/−1-MCP fruit declined faster than the 14.0°C fruit and the 13.0°C/+1-MCP fruit. However, beginning around Day 53, the Y(II) of the 14.0°C/−1-MCP decreased more rapidly, equaling the 13.0°C/−1-MCP treatment over the last week of the experiment.

**Figure 2:**
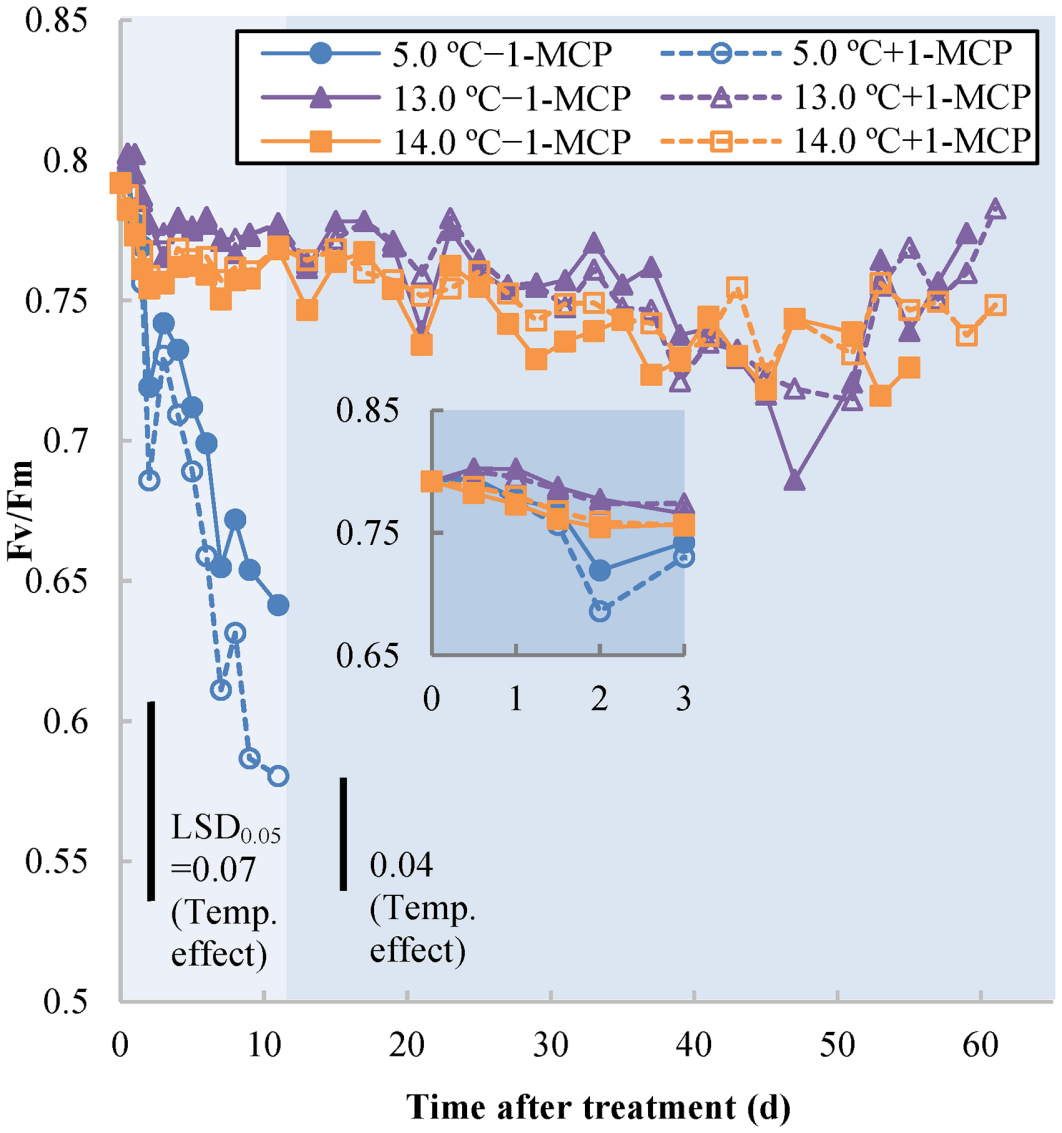
Photochemical efficiency as measured by dark-adapted status photosystem II chlorophyll fluorescence (Fv/Fm) of banana peel initially treated with 0 (−1-MCP) or 50 μL L^−1^ (+ 1-MCP) aqueous 1-MCP for 60 s at 23.0°C and transferred to 5.0, 13.0 or 14.0°C storage with 95% RH (*n* = 6). LSD_0.05_ values varied within different time frames shown by different background shading (days 0–11; days 13–61) due to the various shelf-life durations of treatments.

The other chlorophyll fluorescence indicator, Fv/Fm, represents the maximum photochemical efficiency of PSII in dark-adapted green tissues (Baker, 2008). Similar, to the Y(II) results, the Fv/Fm of fruit stored at 5.0°C (+1-MCP/−1-MCP) decreased from 0.8 to around 0.7 after 1 day, thereafter declining further over the next 10 days, to 0.64 for −1-MCP fruit and 0.58 for +1-MCP fruit (Figure 2). The treatments at 13.0/14.0°C (+1-MCP/−1-MCP) slowly decreased from 0.8 to 0.75 over 45 days. By Day 47, the fruit at 13.0°C briefly declined compared with the 14.0°C fruit, but then rose again to the baseline (0.7–0.75), like the rest of the treatments.

### Electrolyte efflux and malondialdehyde content

Electrolyte efflux indicates the status of membrane function of plant cells in terms of their ability to regulate transport of solutes. Electrolyte efflux of fruit at 5.0°C (+1-MCP/−1-MCP) increased gradually from 15 to 30% during the 31 days of storage while that of fruit at 13.0/14.0°C (+1-MCP/−1-MCP) remained around 13% but increased gradually later during the storage (Table 6). The EE for the −1-MCP treatments at 13.0/14.0°C slightly increased after Day 42; thereafter further increase in EE was more dramatic in the 14.0°C/−1-MCP treatment than in the 13.0°C/−1-MCP treatment. In contrast, the EE of the + 1-MCP treatments at 13.0/14.0°C stayed constant until Day 51, rising only on the last day of storage (Day 61).

**Table 6:** Changes in electrolyte efflux (EE) of banana fruit initially treated with 0 (−1-MCP) or 50 μL L^−1^ (+1-MCP) aqueous 1-MCP for 60 s at 23.0°C and transferred to 5.0, 13.0 or 14.0°C storage with 95% RH (*n* = 6).

Storage temperature (°C)	Treatment	Banana peel electrolyte efflux after 1 day of storage (%)	Days to peel electrolyte efflux above 20%	Banana peel electrolyte efflux (%) at the end of the experiment (day 31 at 5°C; day 61 at 13/14°C)^*^
5.0	−1-MCP	15.07a**	13b	31.15
	+1-MCP	15.74a	10b	28.47
13.0	−1-MCP	14.43a	-	16.39b
	+1-MCP	13.78a	61a	22.55ab
14.0	−1-MCP	14.36a	61a	33.31a
	+1-MCP	15.03a	-	17.10b

* Banana fruit stored at 5.0°C suffered severe chilling injury turning brown and were discarded after 31 days. Before then, there were no ripening characteristics observed.

** Different letters in the same column indicate significant differences by treatments.

Membrane lipid peroxidation resulting from environmental stress can be evaluated by measuring MDA content. The MDA content of banana peel from fruit stored at 5°C was higher than that of the 13/14°C treatments (Supplementary Figure 3) but there was no difference among 1-MCP treatments at 13/14°C. Pulp MDA content is presented in Supplementary Figure 4, showing that similar patterns were shared by all treatments.

## Discussion

Extension of produce shelf-life with retention of high quality is one of the ultimate goals in postharvest handling. Temperature management is a major strategy that is used to reduce metabolic reactions and thus extend shelf life. Another strategy used with climacteric fruits is to delay the onset of the climacteric by avoiding direct ethylene exposure and reducing ethylene production and action by atmosphere control or blockage of ethylene receptors (Watkins, 2006). Temperature management cannot be fully utilized to extend the shelf-life of chilling-sensitive climacteric fruit due to relatively high CTTs that allow ripening to occur while avoiding CI; in the case of banana, shelf life is limited by ripening at the CTT of 14.0°C. Therefore, blocking ethylene action and thus the onset of the climacteric using 1-MCP could be a viable strategy for extending banana shelf life. However, the possibility exists that longer than normal exposure to 14.0°C could result in CI.

Before beginning the experiment, fruit exhibiting any defects, including possible CI symptoms on the peel were discarded. However, CI is cumulative and there remains a possibility that chilling exposure could have occurred in the field or during transportation from Guatemala to Florida without visual symptom development that could be detected by us. There were slight differences in the darkness of the peel green color between lots of bananas, suggesting that some exposure to higher than optimal temperatures may have occurred, but any fruit not initially at stage 2 were discarded.

Banana fruit stored at 5.0°C, whether treated with 1-MCP or not, remained at the initial green, preclimacteric developmental stage during storage, in terms of peel color, texture of peel and pulp, and total pulp soluble solids, until the onset of CI symptoms occurred. The CI symptoms observed at 5.0°C were severe and included inhibition of peel color change, gray epidermal area, vascular browning, and increased stress indicators such as chlorophyll fluorescence [Y(II) and Fv/Fm], EE, and peel MDA content (data not shown) as previous research has indicated (Grierson et al., 1967; Abd El-Wahab and Nawwar, 1977; Chaiprasart et al., 2001; Promyou et al., 2008). Moreover, no obvious 1-MCP effect was observed on fruit at 5.0°C in this study, suggesting no role for ethylene action in CI development under severe chilling temperatures.

Results during storage at 13.0/14.0°C (+1-MCP/−1-MCP) indicated that the delay of the ripening process was affected by both low temperature and 1-MCP. Based on the ripeness stage ratings (Table 1), fruit in the 14.0°C/−1-MCP treatment began to ripen after 34 days, followed by 13.0°C/−1-MCP after 42 days; and 13.0/14.0°C + 1-MCP fruit on Day 51. The ripening rate was affected by both temperature and 1-MCP as the onset of ripening for +1-MCP fruit was delayed by a few more days compared to −1-MCP fruit. Ethylene receptor level has been reported to be the major determinant of initiation of ripening of tomato fruit, and it appears that degradation of ethylene receptors may be induced by ethylene binding, but not 1-MCP binding (Kevany et al., 2007). However, the inhibition of ethylene action by 1-MCP may be alleviated after a certain period of time, and its recovery rate would then be affected by temperature.

Peel color changes, on which the ripeness stage ratings were based, showed minor differences between −1-MCP and + 1-MCP bananas for lightness (L^*^) and chroma or color purity (C^*^), but 1-MCP significantly inhibited changes in h^*^ and also a^*^ value corresponding to loss of green color (Supplementary Figure 5). This 1-MCP effect on color development of banana peel is in agreement with observations that 1-MCP inhibits the breakdown of chloroplasts in most crops (Ku and Wills, 1999; Harris et al., 2000; Mir et al., 2001). However, in the final investigation, there was no obvious difference in peel color at the mild chilling temperature of 13.0°C or the putative lowest safe storage temperature or CTT of 14.0°C, which was supported by Facundo et al. (2015) who reported that mild chilling temperatures (10–13°C) did not affect the color development when cv. Nanicão (AAA type) bananas were fully ripe.

Preclimacteric stage (MG) bananas stored at severe chilling temperature (5.0°C), mild chilling temperature (13.0°C), or CTT (14.0°C) all maintained the same respiration rate and ethylene production rate during storage, which comports with the reports of Gemma et al. (1994) and Sánchez (2016) for chilled bananas; however, Gemma et al. (1994) also observed a burst of respiration and ethylene production after rewarming severely chilled banana fruit (1–5°C) at room temperature. The temperature quotient (Q_10_) is useful to estimate metabolic rates within a known temperature range. The Q_10_ is known to be higher at chilling temperatures for chilling-sensitive crops (Lyons and Breidenbach, 1990). Based on the ethylene production rate measured at 14.0°C in the current study, the expected ethylene production at 5.0°C was estimated using the Q_10_ to be 0.024 ng kg^−1^ s^−1^, but the actual rate measured was 0.04 ng kg^−1^ s^−1^, which was close to the ethylene production rate of the higher temperature treatment. This suggests that there was elevated stress-induced ethylene production at 5.0°C.

The onsets of the climacteric burst in respiration and ethylene production were influenced by the storage temperature and by 1-MCP treatment. Interestingly, there was a 10-day delay between the climacteric rises in respiration and ethylene production for 14.0°C/−1-MCP versus 14.0°C/+1-MCP and 13.0°C/−1-MCP, and another 10-day delay before the climacteric occurred in the 13.0°C/+1-MCP treatment. Peak respiration rates for all treatments at 13.0/14.0°C were similar (5–6 μg kg^−1^ s^−1^) although respiration of +1-MCP fruit at 13.0°C was slightly higher than that of the 13.0°C/−1-MCP fruit. The peak ethylene production of +1-MCP fruit was higher (0.18 ng kg^−1^ s^−1^ at 14.0°C; 0.29 ng kg^−1^ s^−1^ at 13.0°C) than the −1-MCP controls at 13.0/14.0°C (0.12 ng kg^−1^ s^−1^). Delays in the onset of the climacteric along with higher climacteric peaks of respiration and ethylene production in +1-MCP banana fruit were also observed by Golding et al. (1998) and in our previous research (Chang and Brecht, 2020a).

Neither the severe chilling temperature of 5.0°C nor 1-MCP treatment affected the texture of banana peel and pulp during the initial 31-days storage period. In contrast, there was a difference in the 1-MCP effect on texture at 13.0/14.0°C between the peel and pulp tissues. Peel sheer force of −1-MCP fruit at 13.0/14.0°C declined during storage while +1-MCP fruit retained constant shear force throughout the experiment. The softening of the banana fruit pulp at 13.0/14.0°C began after 34 d and proceeded at different rates. The −1-MCP fruit pulp tissue was completely soft (< 2 N) by Day 51 while the pulp of + 1-MCP fruit did not soften to the same firmness until Day 61. Treatment with 1-MCP also delayed softening in papaya fruit (*Carica papaya* L., Hofman et al., 2001; Ergun and Huber, 2004) and was associated with lower pectin methyl esterase (Façanha et al., 2019) and polygalacturonase activities (PG; Fabi et al., 2014) delaying the total pectin degradation (Asmar et al., 2010). Banana softening resulted from the cell wall changes, specifically the synchronized degradation of pectin, hemicellulosic polysaccharides, and starch (Kojima et al.,1994; John and Marchal, 1995). 1-MCP might have affected the banana cell wall degradation during ripening, resulting in the differences among treatments in the current study.

Storage at 13.0°C, just a single degree below the CTT, is a relatively mild chilling stress to banana fruit, and the development of CI symptoms would be expected to develop at a very slow rate. The severity of CI symptoms is determined by a “time × temperature” relationship in which more severe symptoms develop, and develop faster, at lower temperatures and vice versa (Paull, 1990). Therefore, we hypothesized that the CTT of 14.0°C could also be a chilling temperature when the exposure time is extended by inhibiting the shelf-life limiting factor of ripening. Alteration of a few CI symptoms were observed in this study, but more CI symptoms were found to occur at both 13.0°C and 14.0°C if given enough time (Table 6).

Vascular browning was the first CI symptom that appeared, developing earlier in −1-MCP fruit stored at the mild chilling temperature (13.0°C) than at the CTT (14.0°C); +1-MCP delayed but did not prevent the appearance of vascular browning at both of those temperatures. Regarding the external peel appearance related to CI, only fruit held at 5.0°C developed obvious gray skin, but no trace of that CI symptom was found on bananas held at 13.0/14.0°C. Interestingly, when the epidermal layer of the mildly chilled fruit without gray peel was removed, up to 20% of the vascular tissue was affected by vascular browning (data not shown). Therefore, the development of typical peel color in the epidermal layer may obscure mild vascular discoloration. Moreover, vascular browning of +1-MCP fruit at 13.0/14.0°C developed later and with less severity (i.e., lighter browning) than in the control −1-MCP fruit, suggesting involvement of ethylene in vascular browning.

Vascular browning of chilled banana peel has been observed to occur in the laticifer cells, also known as latex vessels, that are associated with vascular bundles (John and Marchal, 1995; Harvey, 2005). Latex in laticifer cells consists of various organelles in a colloidal fluid cytoplasm (Baker et al., 1990). Lutoid vesicles in banana latex may compartmentalize polyphenol oxidase (PPO) and phenols (Kallarackal et al., 1986; Harvey, 2005). Browning reaction of banana fruit results from the oxidation of mainly dopamine by PPO (Griffiths, 1959; Abd El-Wahab and Nawwar, 1977) to produce dopaminequinone-H^+^, which is hypothesized to cooxidize with salsolinol to form salsolinol-*o*-quinone (Sojo et al., 2000). This highly active *o*-quinone would then polymerize to produce melanins as the major browning substance (Castaner et al., 1996). The major enzymes controlling browning in chilling storage were proposed to be phenylalanine ammonia lyase (PAL) and PPO (Nguyen et al., 2003), which are also related to ethylene action in crops (Couture et al., 1993; Pesis et al., 2002; Jin et al., 2011). The interaction of ethylene and those two enzymes at chilling temperature needs to be further investigated.

Chlorophyll fluorescence parameters indirectly reflected the stress status of chlorophyll-containing tissues among different treatments. Quantum yield [Y(II)] is an estimation of PSII operating efficiency (i.e., the rate of electron transport of PSII); Fv/Fm represents the maximal quantum efficiency of PSII photochemistry to detect the loss of function of PSII reaction centers (Öquist et al., 1992; Baker, 2008). The decrease in chlorophyll fluorescence results from the inactivation of the PSII reaction center by stress (e.g., CI) and ripening (breakdown of chloroplasts to form chromoplasts; Chaiprasart et al., 2001) and the inhibition of degreening in 1-MCP-treated fruit may also delay the change of chlorophyll fluorescence (Mir et al., 2001). Of both chlorophyll parameters, Y(II) of 5.0°C fruit decreased rapidly, after only after 0.5 day of storage, followed soon after by a decline in Fv/Fm after 1 day. The chlorophyll fluorescence decline in fruit at 13.0°C after Day 37 might have been due to CI, but the subsequent drop of the 14.0°C/−1-MCP fruit may have been a result of ripening-induced chlorophyll breakdown. The early CI stress status of 13.0°C fruit was also detectable from Y(II) and occurred before the onset of ripening. Therefore, Y(II) could be an appropriate tool to examine early CI stress on the MG banana.

Chilling temperature may lead to alteration of the physical properties of lipids in plant membrane systems (Lyons, 1973), possibly resulting in unbalanced metabolism that leads to lipid peroxidation and accumulation of reactive oxygen species (ROS; Zhang et al., 2005; Nukuntornprakit et al., 2015). Therefore, EE and MDA content are used as essential markers to evaluate membrane permeability and peroxidation, respectively, as indicators of membrane integrity. Malondialdehyde content, one parameter of membrane integrity, showed the temperature effect but no significant 1-MCP effect (Supplementary Figures 3, 4). However, changes in EE and MDA can be affected by ripening and senescence as well as by CI (Gemma et al., 1994; Lim et al., 2007), all of which lead to breakdown of membrane systems. Increased EE in bananas at 5.0°C (+1-MCP/−1-MCP) coincided with the appearance and severity of CI symptoms, including vascular browning and the reduction in chlorophyll fluorescence [Y(II) and Fv/Fm]. In contrast, the changes of EE at 13.0/14.0°C (+1-MCP/−1-MCP) followed the progress of ripening as shown by changes in peel color, and fruit respiration rate, ethylene production, and texture.

Compared to the sensitive CI response of banana peel, the pulp appears to be less affected by chilling temperature based on texture. The changes were delayed by temperature and 1-MCP, but recovered as storage progressed. The difference in chilling sensitivity between banana peel and pulp was also observed by Gemma et al. (1994) in terms of the patterns of EE at different temperatures in the two tissues, which showed breakpoints at 8.9 and 3.0°C, respectively, indicating a similar conclusion that the peel of bananas is more sensitive to CI than the pulp.

## Conclusion

Chilling threshold temperature is a limitation for postharvest handling of climacteric tropical fruit like banana in that CI is avoided but ripening may occur at the CTT. This research demonstrates that this putative safe temperature may become a *de facto* chilling temperature if the shelf-life-limiting factor is removed, allowing longer exposure. Longer exposure time at the reported banana fruit CTT of 14.0°C (19 days for −1-MCP treatment; 25 days for +1-MCP treatment) caused relatively mild CI on the fruit before recovery from 1-MCP-induced ripening inhibition occurred. Vascular browning was the most sensitive indicator of CI status while Y(II) was also determined to be a potential non-destructive tool to detect early CI stress in MG banana. 1-MCP-treated fruit at 13.0/14.0°C developed less vascular discoloration, less EE, and higher quantum yield [Y(II)] than fruit without 1-MCP, but 1-MCP did not reduce development of external peel discoloration or affect Fv/Fm at the same temperatures, which suggests that ethylene might be involved in early development of some, but not all CI symptoms.

## Data availability statement

The raw data supporting the conclusions of this article will be made available by the authors, without undue reservation.

## Author contributions

L-YC and JB conceived and designed the experiments, analyzed the data, and wrote the manuscript. L-YC performed the experiments. L-YC, SS, JK, and JB reviewed and revised the manuscript. All authors contributed to the article and approved the submitted version.

## Funding

L-YC was sponsored by the Elite program of the Council of Agriculture, Taiwan for her Ph.D. The authors declare that this study received funding from It’s Fresh! Ltd. The funder was not involved in the study design, collection, analysis, interpretation of data, the writing of this article, or the decision to submit it for publication.

## Conflict of interest

The authors declare that the research was conducted in the absence of any commercial or financial relationships that could be construed as a potential conflict of interest.

## Publisher’s note

All claims expressed in this article are solely those of the authors and do not necessarily represent those of their affiliated organizations, or those of the publisher, the editors and the reviewers. Any product that may be evaluated in this article, or claim that may be made by its manufacturer, is not guaranteed or endorsed by the publisher.

## Supplementary material

The Supplementary material for this article can be found online at: https: https://www.frontiersin.org/articles/10.3389/fpls.2022.966789/full#supplementary-material
