# Supplementary material for: Delaying ripening using 1-MCP reveals chilling injury symptom development at the putative chilling threshold temperature for mature green banana
Source: Front Plant Sci. 2022 Sep 15;13:966789. doi: 10.3389/fpls.2022.966789 (PMC9515583; doi:10.3389/fpls.2022.966789)
Supplement: Supplementary file 1 [file Data_Sheet_1.docx]

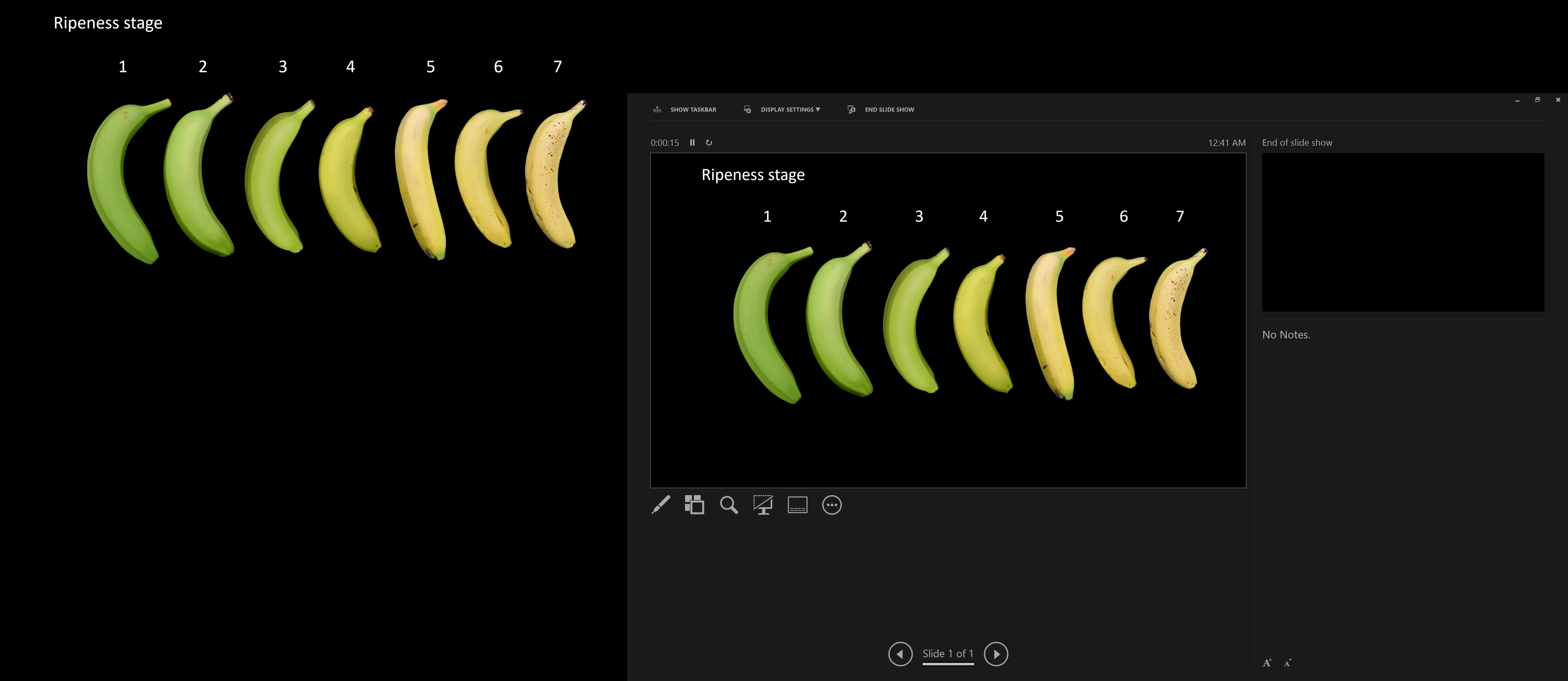


**Supplementary Figure 1**. Appearance of normal ripening bananas in this study from stage 1 (dark green) to stage 7 (fully yellow with brown spots).


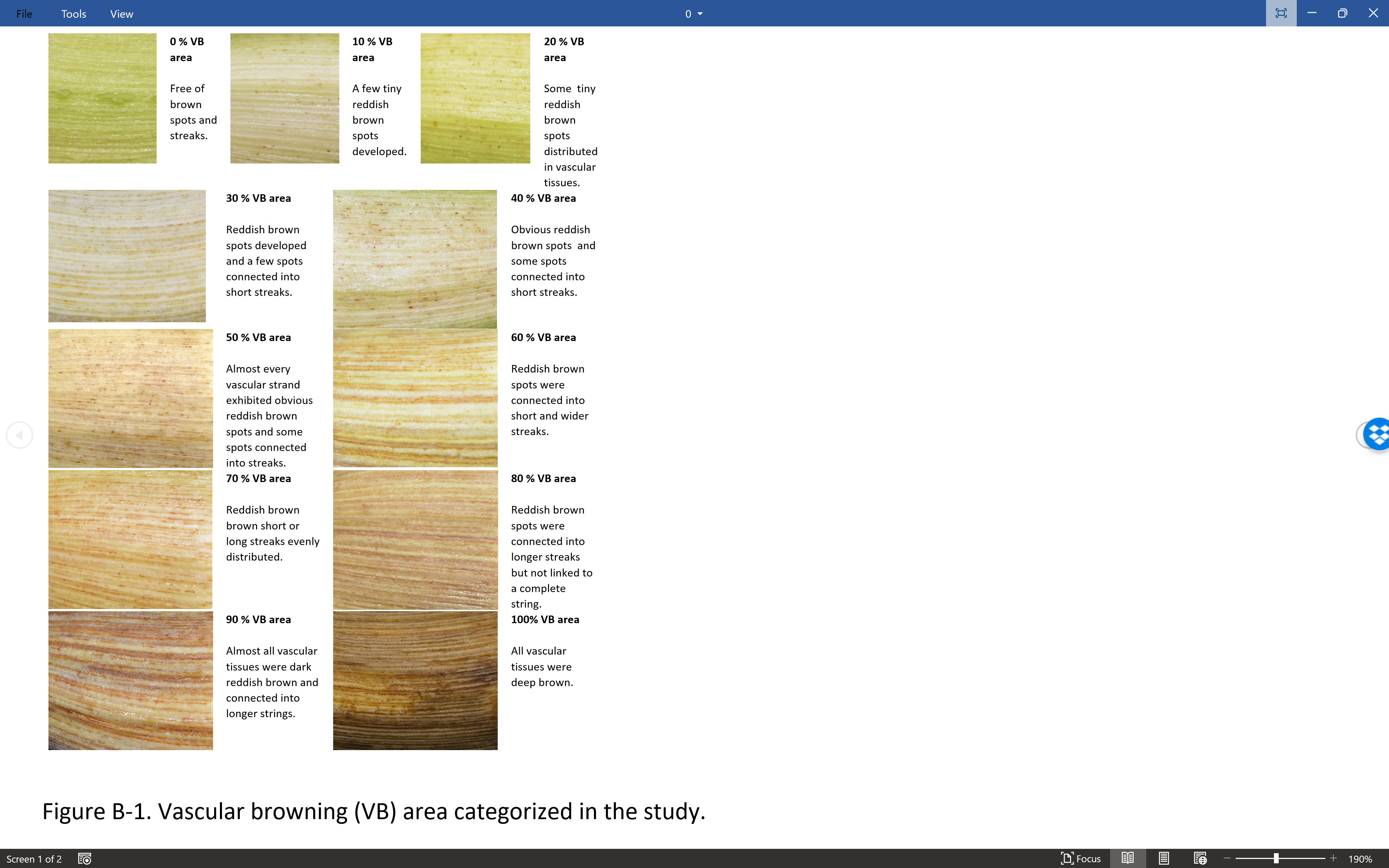


**Supplementary Figure 2**. Vascular browning (VB) area categorized in the study.

0.008

(1-MCP effect)

LSD_0.05_

=0.015

(Temp.

effect)

**Supplementary Figure 3**. MDA content of banana peel initially treated with 0 or 50 µL L^-1^ aqueous 1-MCP for 60 s at 23.0 °C and transferred to 5.0, 13.0 or 14.0 °C storage with 95 % RH (n = 6). LSD_0.05_ values varied within different time frames shown by different background shading (Days 0 - 31; Days 37 - 61) due to the various shelf-life durations of treatments.

LSD_0.05_

=0.03

(Temp.

×

1-MCP)

n.s.

**Supplementary Figure 4**. MDA content of banana pulp initially treated with 0 or 50 µL·L^-1^ aqueous 1-MCP for 60 s at 23.0 °C and transferred to 5.0, 13.0 or 14.0 °C storage with 95 % RH (n = 6). LSD_0.05_ values varied within different time frames shown by different background shading (Days 0-31; Days 37-61) due to the various shelf-life durations of treatments.

(A) (B)

LSD_0.05_

=0.78

(Temp.

effect)

0.99

(1-MCP effect)

1.51

(both factors)

LSD_0.05_

=0.54

(Temp.

effect)

**Supplementary Figure 5**. Peel color changes of banana fruit initially treated with 0 (−1-MCP) or 50 µL L^-1^ (+1-MCP) aqueous 1-MCP for 60 s at 23.0 °C and transferred to 5.0, 13.0 or 14.0 °C storage with 95 % RH in terms of (A) a* value and (B) b* value (n = 3). LSD0.05 values within different time frames shown by different background shading (Days 0 - 31; Days 33 - 61) due to the various shelf-life durations of treatments.
