# Supplementary figures and images for: Delaying ripening using 1-MCP reveals chilling injury symptom development at the putative chilling threshold temperature for mature green banana
Source: Front Plant Sci. 2022 Sep 15;13:966789. doi: 10.3389/fpls.2022.966789 (PMC9515583; doi:10.3389/fpls.2022.966789)

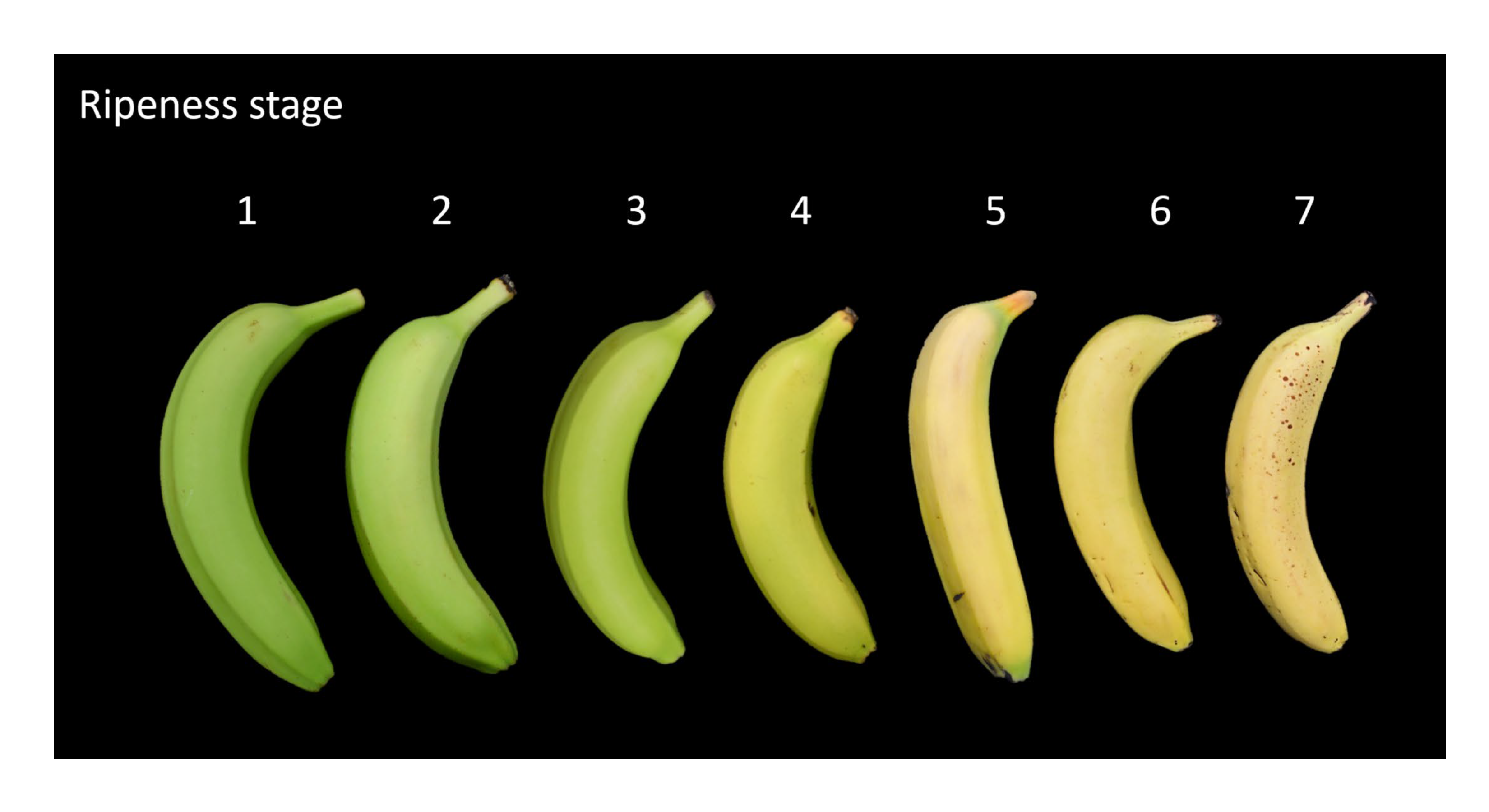

Supplement: Supplementary file 2 [file Image_1.TIF]

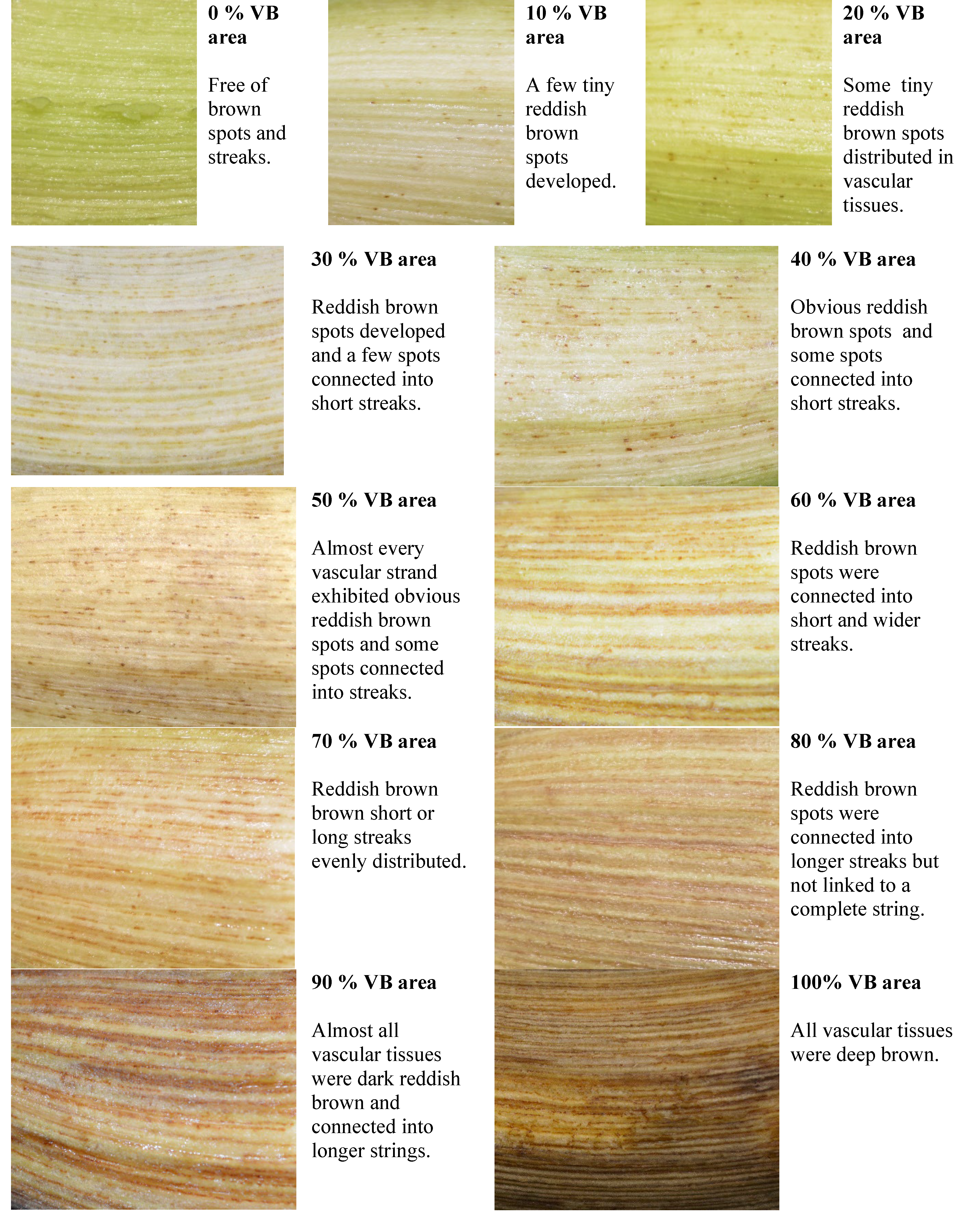

Supplement: Supplementary file 3 [file Image_2.TIF]

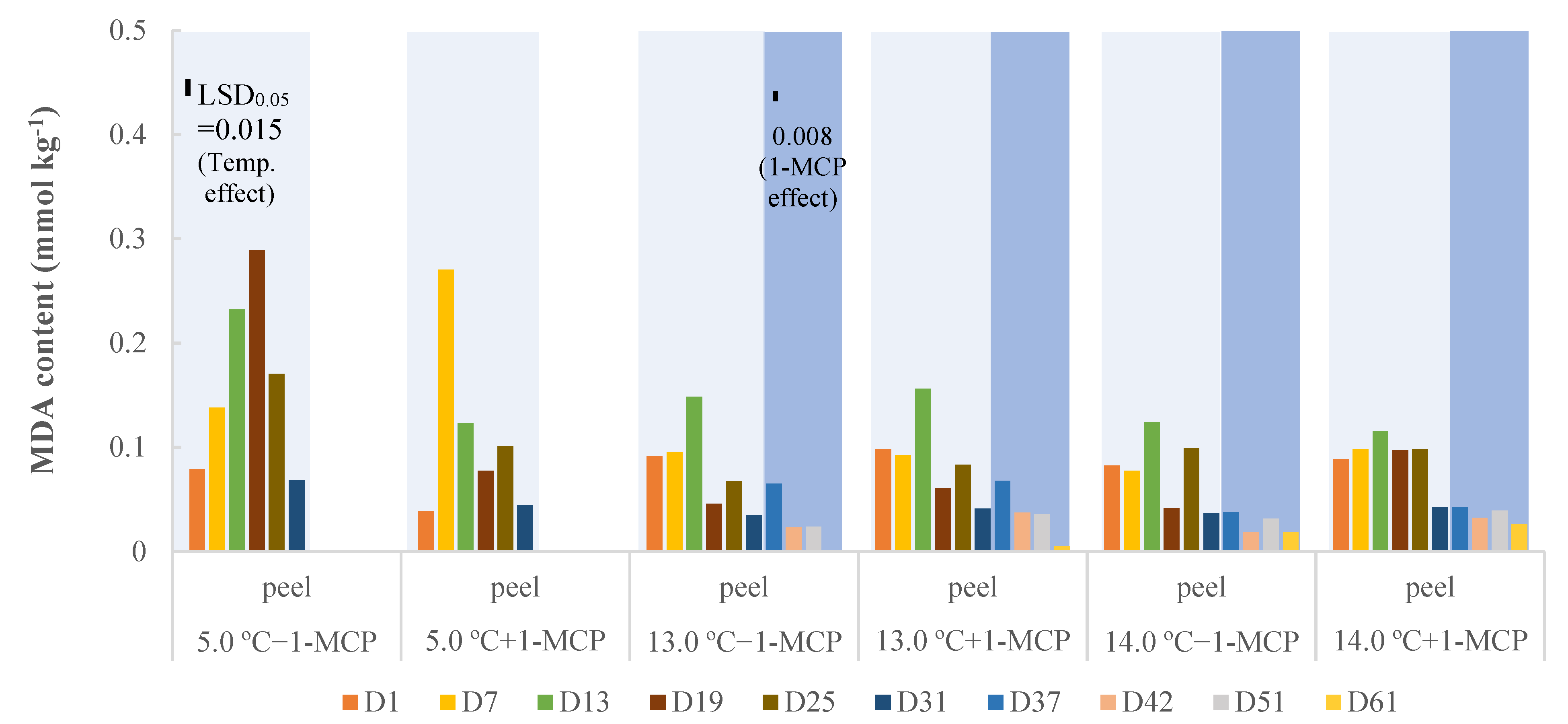

Supplement: Supplementary file 4 [file Image_3.TIF]

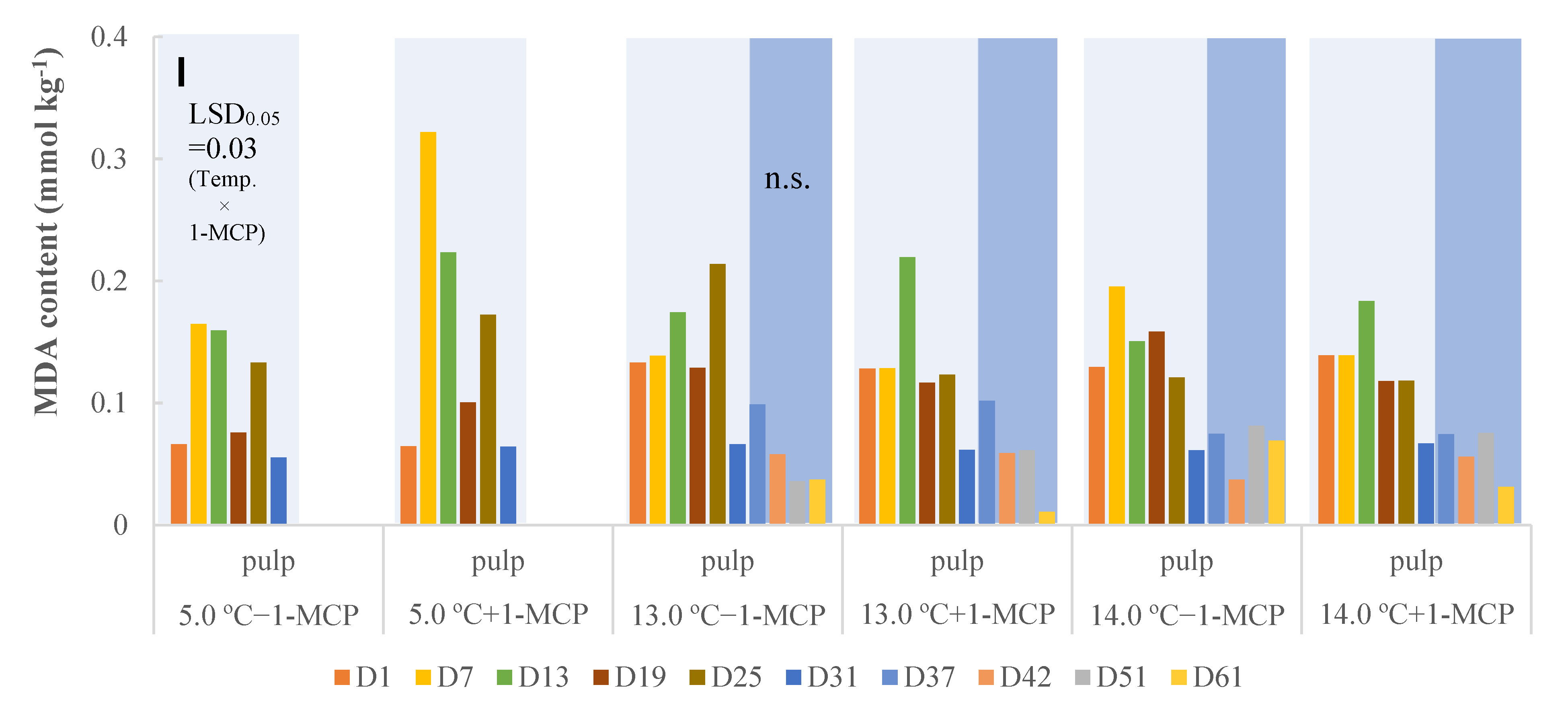

Supplement: Supplementary file 5 [file Image_4.TIF]

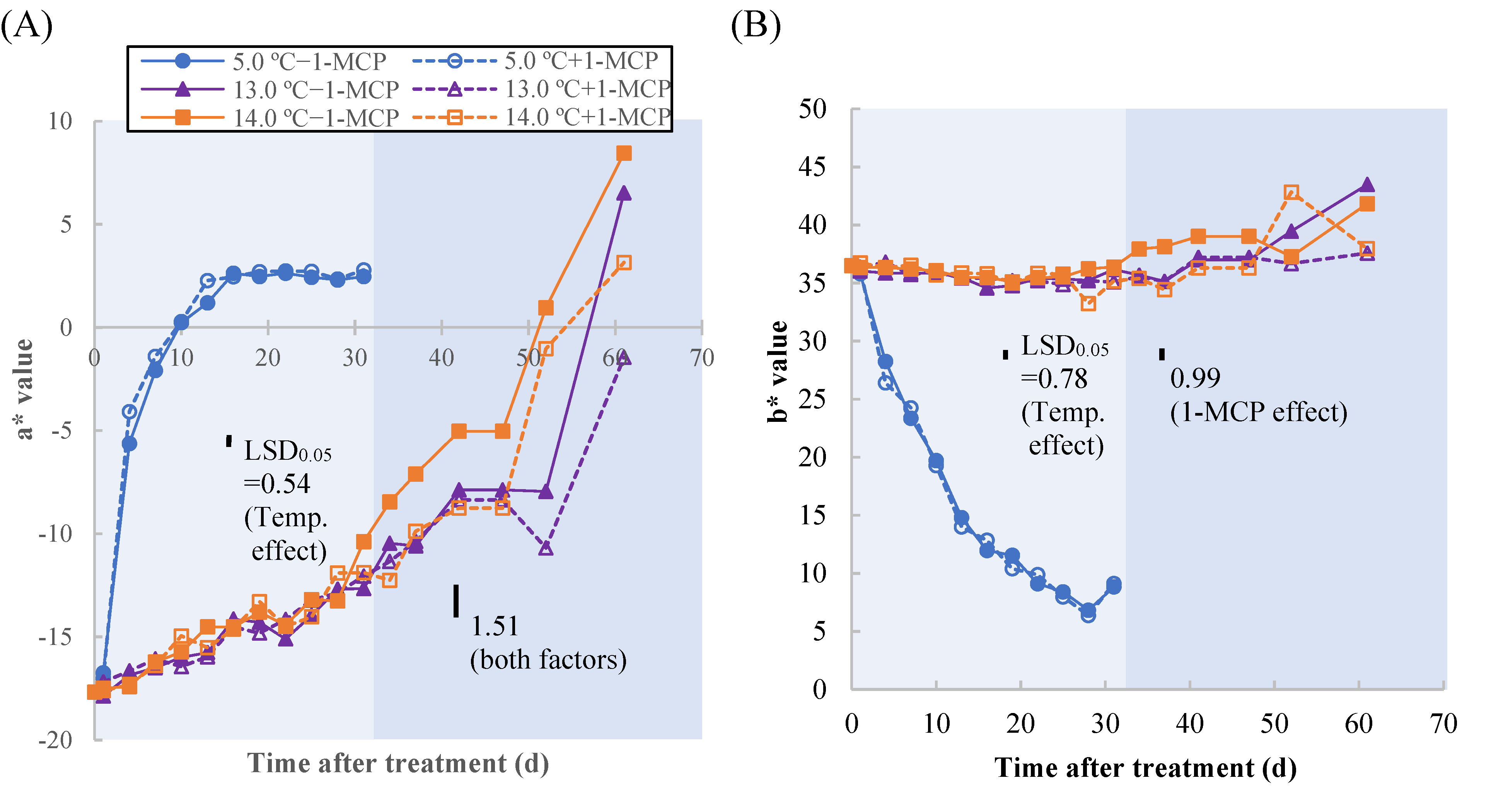

Supplement: Supplementary file 6 [file Image_5.TIF]
